# Animal-type melanoma/pigmented epithelioid melanocytoma: three clinical cases of a rare and controversial entity^☆^

**DOI:** 10.1016/j.abd.2022.06.007

**Published:** 2023-06-29

**Authors:** Vittorio Gedda, Francisco González-Coloma, Alejandro Jeldres, Carolyn Rodríguez, Gabriela Coulon, Alex Castro

**Affiliations:** a Department of Dermatology, Faculty of Medicine, Universidad de Chile, Santiago, Chile; b Health Reference Center Peñalolén Cordillera Oriente, Metropolitano Oriente Health Service, Santiago, Chile; c Santiago Oriente Hospital Dr. Luis Tisné Brousse, Metropolitano Oriente Health Service, Santiago, Chile; d Department of Surgery, Dermatology Service, Clínica Alemana, Universidad del Desarrollo, Santiago, Chile; e Department of Pathology, Clínica Alemana, Universidad del Desarrollo, Santiago, Chile

Dear Editor,

Animal-type melanoma (ATM) is an infrequent variety of melanoma with a great diversity of nomenclatures, such as “equine-type melanoma”, “pigment synthesizing melanoma”, “epithelioid pigmented melanocytoma” or simply “animal melanoma”.^1^ Also is a controversial entity regarding its histopathological characterization,^2^ what forced the last classification of skin tumors by the World Health Organization (WHO), published in 2018, to achieve a convergence under the term “pigmented epithelioid melanocytoma” (PEM).^3^ Clinical cases of PEM are presented, which correspond to the totality of diagnosed cases between 2018 and 2021 in 7 public and private health reference centers in Santiago of Chile.

## Case 1

**Figure 1:**
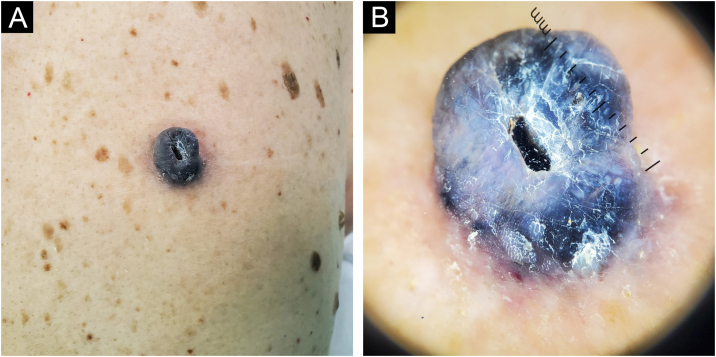
Case 1. (A) Intensely pigmented exophytic tumor with ulcerated center in the lumbar region. (B) Dermatoscopy shows a homogeneous blue-violet pattern, blue-grey veil, and central ulceration

**Figure 2:**
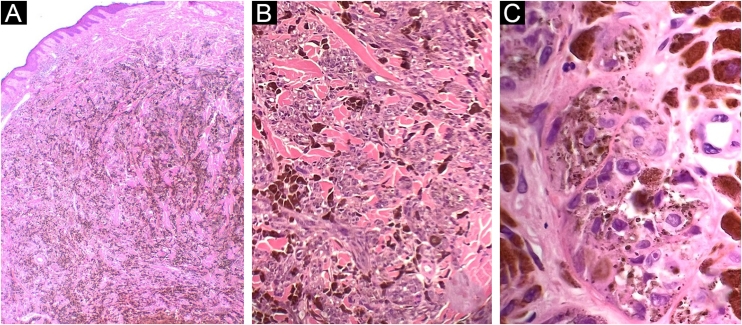
Case 1. (A) Dermal melanocytic neoplasm, with abundant amount of pigment and preserved epidermis (Hematoxylin & eosin, ×2). (B) Nests of atypical melanocytes intermingled with abundant melanophages and collagen bundles (Hematoxylin & eosin, ×10). (C) Epithelioid and fusiform melanocytes, with accentuated atypia and macronucleoli (Hematoxylin & eosin, ×40)

83-year-old man, with two previously resected spinocellular carcinomas, presented a black-blue tumor of 5 years of evolution on the lumbar region, painful and ulcerated, which slowly grew to 3 cm diameter at diagnosis (Fig. 1A). Without palpable lymphadenopathies. Dermoscopy showed a homogeneous blue-violet pattern, blue-gray veil, and structureless whitish areas (Fig. 1B). Histopathology reported an infiltrating ATM, with epithelioid and fusiform dermal melanocytes, abundant pigment, accentuated atypia, Clark's level V, 9.5 mm of Breslow, 0 to 1 mitosis/mm,^2^ without ulceration, without vascular or perineural invasion (Fig. 2). Positron Emission Tomography (PET) revealed a solid hypermetabolic mass in the superior lobe of the right lung, without nodal or visceral metastasis. The surgical team performed the wide local excision (WLE) with sentinel lymph node biopsy, resulting negative, and confirmed that the pulmonary mass was a lung adenocarcinoma, with survival after 6 months of follow-up.

## Case 2

**Figure 3:**
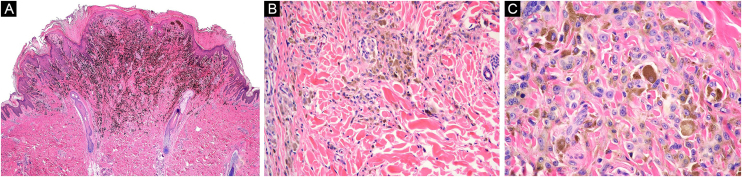
Case 2. (A) Wedge-shaped melanocytic proliferation at the dermal-epidermal junction and superficial dermis, intensely pigmented (Hematoxylin & eosin, ×2). (B) Epithelioid and dendritic melanocytic cells arranged in nests, some pigmented, with signs of maturation towards depth (Hematoxylin & eosin, ×10). (C) Neoplastic cells with wide cytoplasm, abundant granular brown melanin pigment, and slightly hyperchromatic and pleomorphic nuclei, with prominent nucleoli (Hematoxylin & eosin, ×40)

A 16-year-old healthy man presented a blackish nodule in the right flank, 3 years of slow growth, asymptomatic, 7 mm diameter, with well-defined edges and smooth surface. Dermoscopy showed a homogenous bluish-black pattern, shiny-white structures, and a faint pigmentary net in its proximal margin. Histopathology reported a PEM, showing epithelioid and dendritic melanocytes with vesicular nuclei and wide cytoplasm with abundant granular brown pigment, arranged in nests and isolated units in the dermo-epidermal junction and dermis, preserved maturation, and numerous melanophages (Fig. 3). Oncological committee indicated WLE and clinical follow-up, with survival after 12 months.

## Case 3

**Figure 4:**
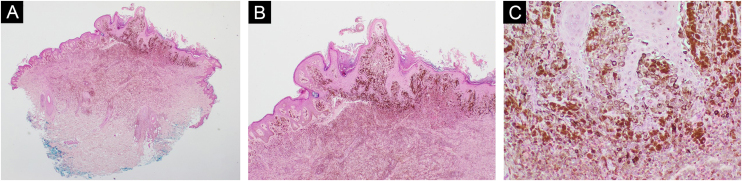
Case 3. (A and B) Symmetrical proliferation of pigmented epithelioid melanocytes in reticular and superficial dermis, arranged in nests and short bundles with preserved maturation (Hematoxylin & eosin, ×2 and ×4). (C) Pigmented epithelioid melanocytes with mild atypia (Hematoxylin & eosin, ×10)

A 7-year-old healthy boy presented a brown blotch in the lumbar region, asymptomatic, with well-defined edges and verrucous surface, 1 year of fast growth, with a diameter of 1 cm at diagnosis. Dermoscopy showed a homogeneous black pattern, without asymmetry in color or shape. Histopathological reported a PEM, whose dermal epithelioid melanocytes presented mild atypia, preserved maturation, with focal presence in the dermo-epidermal junction arranged in nests and short bundles, with focal extension to appendages, blood vessels, and nerves (Fig. 4). The medical team performs a WLE and clinical follow-up, with survival after 8 months.

The limited number of cases found confirms the low frequency of this pathology, with only isolated clinical cases and series of cases, except for a meta-analysis with a total of 190 cases.^1^ PEM tends to occur in young patients, mean of 27 years, with the same proportion between genders and a lower prevalence in Caucasian versus other skin cancers. Clinically, they usually presented a blue-blackish blotch or nodule, with occasional ulceration, located preferably in extremities.^1^, ^4^ Dermoscopic findings are homogeneous black, brown, or bluish patterns, shiny-white structures, blue-white veils, and irregular white areas.^5^

In Chile, a series of four patients ATM was published, mostly men, with mean of 46 years, with a Breslow index between 1.5 and 14 mm, with nodal compromise in half of the cases, and all with a survival greater than 5 years.^6^ In Argentina, there is a published case of a 31-year-old male with an ATM with a Breslow index of 2.1 mm, positive sentinel node, and survival greater than 5 years.^7^ In Brazil, a report of two cases of ATM, a 49-year-old man and a 32-year-old woman, whose histopathological analyses present discrete nuclear atypia and few mitoses.^8^ In Colombia, a 41-year-old man with ATM was reported, performing a WLE without sentinel lymph node biopsy.^9^

Histologically, a spectrum of findings is described. The most characteristic elements are the presence of fusiform, dendritic and/or epithelioid melanocytes, with a periadnexal growth pattern, an intensely pigmented cytoplasm, vesicular nuclei, prominent nucleoli, and melanophages.^2^ They may present moderate to severe atypia, being habitual a mitotic index superior to 2× mm.^2^ A distinction can be made according to whether it corresponds to a pure injury (pure PEM) or associated with more conventional components of nevus (combined PEM).^4^ In the cases shown in this series it is observed the ends of the finding spectrum make complex the diagnosis of PEM/ATM; from the first case in an aged patient, with previous skin malignancies, an alarming clinical, and accentuated cellular atypia; to other two cases of pediatric patients, healthy, with a suggestive clinical that motivates the removal and mild cellular atypia.

Despite the latest consensus of the WHO, some authors propose histological clues suggestive of ATM, such as a background solar damage, atypical pagetoid component, severe nuclear atypia, expansive growth, and mitotic activity greater than 2× mm.^2^, ^4^ Regarding genetic studies, pure PEM presents a fusion of the PRKCA gene, whereas combined PEM commonly have mutations in the BRAF and PRKAR1A genes, and may be present in pigment synthesizing melanomas, for whose distinction other molecular techniques such as Fluorescent In Situ Hybridization (FISH) or Comparative Genomic Hybridization (CGH) become relevant.^4^

Surgical management is preferred, opting for a WLE with or without a sentinel node in 99.2% of cases.^1^ At diagnosis, only 2.1% of patients presented palpable lymph nodes and only 1% had distant metastases,^1^ but in a series of patients where sentinel node was performed there was a positivity between 46% and 60%.^6^, ^10^ There is consensus regarding the non-aggressive behavior of the PEM, but it is required studies that carry out long-term follow-up of these patients.

ATM/PEM continues to be a controversial entity, in which histopathological diagnosis is not only a matter of nomenclature but also affects subsequent clinical behavior, strengthening the importance of the clinical-pathological relationship. It’s urgent to establish a consensus regarding the management and monitoring of these patients, to develop future studies that allow us to continue understanding their biological behavior.

## Financial support

None declared.

## Authors’ contributions

Vittorio Gedda: Study concept and design; Data collection, or analysis and interpretation of data; Writing of the manuscript or critical review of important intellectual content; Data collection, analysis and interpretation; Effective participation in the research guidance; Critical review of the literature; Final approval of the final version of the manuscript.

Francisco González-Coloma: Study concept and design; Data collection, or analysis and interpretation of data; Writing of the manuscript or critical review of important intellectual content; Data collection, analysis and interpretation; Effective participation in the research guidance; Intellectual participation in the propaedeutic and/or therapeutic conduct of the studied cases; Critical review of the literature; Final approval of the final version of the manuscript.

Alejandro Jeldres: Study concept and design; Data collection, or analysis and interpretation of data; Data collection, analysis and interpretation; Effective participation in the research guidance; Intellectual participation in the propaedeutic and/or therapeutic conduct of the studied cases; Final approval of the final version of the manuscript.

Carolyn Rodríguez: Data collection, or analysis and interpretation of data; Data collection, analysis and interpretation; Intellectual participation in the propaedeutic and/or therapeutic conduct of the studied cases; Final approval of the final version of the manuscript.

Gabriela Coulon: Data collection, or analysis and interpretation of data; Data collection, analysis and interpretation; Intellectual participation in the propaedeutic and/or therapeutic conduct of the studied cases; Final approval of the final version of the manuscript.

Alex Castro: Data collection, or analysis and interpretation of data; Data collection, analysis and interpretation; Final approval of the final version of the manuscript.

## Conflicts of interest

None declared.
